# “Emergency Room Evaluation and Recommendations” (ER2) Tool for the Screening of Older Emergency Department Visitors With Major Neurocognitive Disorders: Results From the ER^2^ Database

**DOI:** 10.3389/fneur.2021.767285

**Published:** 2022-01-06

**Authors:** Olivier Beauchet, Liam A. Cooper-Brown, Joshua Lubov, Gilles Allali, Marc Afilalo, Cyrille P. Launay

**Affiliations:** ^1^Department of Medicine, University of Montreal, Montreal, QC, Canada; ^2^Research Center of the Geriatric University Institute of Montreal, Montreal, QC, Canada; ^3^Division of Geriatric Medicine, Department of Medicine, Sir Mortimer B. Davis Jewish General Hospital, Lady Davis Institute for Medical Research, McGill University, Montreal, QC, Canada; ^4^Lee Kong Chian School of Medicine, Nanyang Technological University, Singapore, Singapore; ^5^Faculty of Medicine, McGill University, Montreal, QC, Canada; ^6^Division of Neurology, Department of Neurosciences, Geneva University Hospitals, Geneva, Switzerland; ^7^Leenaards Memory Center, Lausanne University Hospital, University of Lausanne, Lausanne, Switzerland; ^8^Emergency Department, Jewish General Hospital, McGill University, Montreal, QC, Canada

**Keywords:** emergency department, major neurocognitive disorders, screening, older adults, performance evaluation

## Abstract

**Purpose:** The Emergency Room Evaluation and Recommendation (ER^2^) is an application in the electronic medical file of patients visiting the Emergency Department (ED) of the Jewish General Hospital (JGH; Montreal, Quebec, Canada). It screens for older ED visitors at high risk of undesirable events. The aim of this study is to examine the performance criteria (i.e., sensitivity, specificity, positive predictive value [PPV], negative predictive value [NPV], positive likelihood ratio [LR+], negative likelihood ratio [LR-] and area under the receiver operating characteristic curve [AUROC]) of the ER^2^ high-risk level and its “temporal disorientation” item alone to screen for major neurocognitive disorders in older ED visitors at the JGH.

**Methods:** Based on a cross-sectional design, 999 older adults (age 84.9 ± 5.6, 65.1% female) visiting the ED of the JGH were selected from the ER^2^ database. ER^2^ was completed upon the patients' arrival at the ED. The outcomes were ER^2^'s high-risk level, the answer to ER^2^'s temporal disorientation item (present vs. absent), and the diagnosis of major neurocognitive disorders (yes vs. no) which was confirmed when it was present in a letter or other files signed by a physician.

**Results:** The sensitivities of both ER^2^'s high-risk level and temporal disorientation item were high (≥0.91). Specificity, the PPV, LR+, and AROC were higher for the temporal disorientation item compared to ER^2^'s high-risk level, whereas a highest sensitivity, LR-, and NPV were obtained with the ER^2^ high-risk level. Both area under the receiver operating characteristic curves were high (0.71 for ER^2^'s high-risk level and 0.82 for ER^2^ temporal disorientation item). The odds ratios (OR) of ER^2^'s high-risk level and of temporal disorientation item for the diagnosis of major neurocognitive disorders were positive and significant with all OR above 18, the highest OR being reported for the temporal disorientation item in the unadjusted model [OR = 26.4 with 95% confidence interval (CI) = 17.7–39.3].

**Conclusion:** Our results suggest that ER^2^ and especially its temporal disorientation item may be used to screen for major neurocognitive disorders in older ED users.

## Introduction

Major neurocognitive disorders are common in older emergency department (ED) visitors, with a prevalence up to 30% (1–4). These disorders are associated with a high risk of undesirable events. For instance, older Canadian patients with major neurocognitive disorders are 1.5 times more likely to experience undesirable events, such as delirium, falls and, prolonged length of stay, compared to their cognitively healthy counterparts (5). The presence of major neurocognitive disorders is often unknown upon arrival to the ED (1–3). However, detecting major neurocognitive disorders early is helpful to initiate the appropriate management and prevention strategies (4, 5). Therefore, screening for major neurocognitive disorders in older ED patients may limit the occurrence of delirium or other undesirable hospital-related events and is thus recommended early in the hospital care process (4).

Hospital admission is often preceded by an ED visit in older adults (6–8). Therefore, screening for major neurocognitive disorders should systematically be included as a step in assessing older patients upon arrival to the ED. There are obstacles to implementing systematic screenings of major neurocognitive disorders in EDs (9, 10). First, EDs are under duress because of overcrowding, delays, and diversions, which have increased to epidemic proportions (6). In Canada, these ED features are exacerbated by the scarce number of hospital beds per capita, which is one of the lowest among Organization for Economic Cooperation and Development (OECD) nations^1^. Second, EDs are configured for the care of single acute diseases, rather than chronic diseases associated with disabilities like Alzheimer's disease, the most common cause of major neurocognitive disorders (6, 7). Third, human resources, including the experience and demands on time of ED staff, represent a practical barrier to completing cognitive assessments (3, 4). Fourth, the prevalence of undiagnosed Alzheimer's disease amongst older patients may be as high as 90% (11).

The screening of major neurocognitive disorders needs to be compatible with the daily practice of ED staff. The Emergency Room Evaluation and Recommendations (ER^2^) tool is a validated clinical assessment that screens for older ED users at a high risk of undesirable events (12, 13). The tool is composed of 6 items (age, sex, use of home support, polypharmacy, use of walking aid and inability to give month and/or year), providing a score ranging from 0 to 14 and classifying patients into three risk levels: low, moderate, and high. The inability to give month and/or year is the only ER^2^ item which explores cognition. Temporal disorientation is frequently affected at advanced stages of neurocognitive disorders and, thus, this item could be used to screen individuals with undiagnosed major neurocognitive disorders, regardless of their etiology (3, 4, 9, 11). Since 2017, ER^2^ has been included in the electronic medical record of ED patients at the Jewish General Hospital (JGH, Montreal, Quebec, Canada) as part of clinical quality improvement program. In this hospital, ER^2^ is used by ED nurses like a application to assess older ED users on stretchers upon their arrival. Its completion takes < 3 min, after which the ER^2^ score and risk level are automatically provided (12). Thus, ER^2^ is a quick assessment which is compatible with the ED daily practice usually characterized by a huge workflow. In addition, ER^2^ helps ED staff to early consider the most relevant geriatric syndromes in order to make the best choices for the older ED patients (12, 13). ER^2^ is, thus, used as a triage tool for older ED users. All information collected from the electronic medical record of older ED users is recorded in a database with the agreement of the Research Review Office of the Jewish General Hospital and the Research Ethics Committee of the Integrated Health and Social Services University Network for West-Central Montreal (Montreal, Quebec, Canada). The ER^2^ database represents an opportunity to test the hypothesis that the ER^2^ “high” risk level and/or its “temporal disorientation” item may be used to screen older ED users with major neurocognitive disorders. The aim of this study was to examine the performance criteria (i.e., sensitivity, specificity, positive predictive value [PPV], negative predictive value [NPV], positive likelihood ratios [LR+], negative likelihood ratios [LR-] and area under the receiver operating characteristic curve [AUROC]) of the ER^2^ high risk level and the temporal disorientation item alone for screening major neurocognitive disorders in older ED users at the JGH.

## Methods

### Study Design and Population

Based on a cross-sectional design, 999 older adults (age 84.9 ± 5.6, 65.1% female) visiting the ED of the JGH (Montreal, Quebec, Canada) were selected from the ER^2^ database. The inclusion criteria were age ≥ 75, unplanned ED visit, being on a stretcher, ER^2^ information collected and score recorded, and patient agreement for data recording in the ER^2^ database. The selected participants were patients who consecutively visited the JGH ED in the first year (September 2017 to September 2018) of collecting information for the ER^2^ database.

### Emergency Room Evaluation and Recommendations Tool

ER^2^ is a component of ED patients' electronic medical record. It is used as an application to automatically calculate the ER^2^ score and level of risk for undesirable hospital-related events (12, 13). Six close-ended questions (i.e., yes vs. no) compose ER^2^. They are: age 85 and over, male, polypharmacy (defined as number of medications taken daily ≥5), use of formal (i.e., health care or social services) and/or informal (i.e., family and/or friend) home support, use of a walking aid regardless of type, and temporal disorientation (defined as inability to name the current month and/or year). A score of five points is assigned to items “use of a walking aid” and “temporal disorientation,” while a score of one point is assigned to the other items. The ER^2^ score ranges from 0 (lowest risk) to 14 (highest risk) and stratifies risk for undesirable events into low (score 0–3), medium (score 4–5) and high (score ≥6) risk levels (12).

### Assessment of Participant Characteristics

Participants were assessed by the triage nurse upon their arrival to the ED of the JGH. Information including patients' age, sex, and place of living was thereby collected and recorded in the electronic medical record, as was the Canadian ED Triage and Acuity Scale (14). This scale classifies patients according to the type and urgency of their health condition for prioritization purposes. It is composed of 5 levels of urgency: level 1, defined as resuscitation; level 2, defined as emergent; level 3, defined as urgent; level 4, defined as less urgent; and level 5, defined as non-urgent. Reasons for ED visits were also recorded in the patients' files. In this study, this information was classified into 5 sub-types: Organ failure, defined as an acute organ decompensation; mobility disorders, defined as gait and/or balance impairment and/or fall with or without fall-related injuries; Neuropsychiatric disorders, defined as delirium, dementia, and/or behavioral disorders; A social issues, defined as the absence of symptoms of acute disease and combined with an acute increase in the use of formal and/or informal home and social services leading to an inability to cope with life circumstances. Once triage was completed and the older ED user was on a stretcher, a designated nurse completed ER^2^ at bedside.

For this study, information on major neurocognitive disorders was collected through a review of the patient's digital files by two research assistants. The diagnosis of major neurocognitive disorders was confirmed when it was present in a letter or other files signed by a physician.

### Outcomes

The outcomes were the ER^2^ “high” risk level, the answer to ER^2^'s “temporal disorientation” item (present vs. absent) and the diagnosis of major neurocognitive disorders (yes vs. no).

### Participant Consent and Protocol Approval and Registration

Verbal informed consent was obtained for all participants following a systematic and standardized process used in the ED ward of the JGH. Participants or their legal guardian, when appropriate, were informed that their medical information may be used for research purposes. If they disagreed, they informed the treating physician, and a note was recorded in their chart. The Ethics Committee the Jewish General Hospital approved this process.

### Statistical Analysis

Two research assistants independently conducted data extraction. A consensus procedure was defined in case of disagreement but was not implemented due to concordance. The participant's baseline characteristics were summarized using means, standard deviations (SD), frequencies and percentages, as appropriate. They were separated into two groups: participants with major neurocognitive disorders and those without. Chi-square or unpaired *t*-tests were used to compare groups. The performance criteria (i.e., sensitivity, specificity, PPV, NPV, positive and negative LR and AUROC) of the ER^2^ high risk level and temporal disorientation item alone for screening major neurocognitive disorders were calculated.

Two separate logistic regression models were constructed to examine the association between major neurocognitive disorders used as dependent variable and the ER^2^ high risk level and the ER^2^ temporal disorientation item used as independent variables. *P*-values < 0.05 were considered statistically significant. All statistics were performed using SPSS (version 23.0; SPSS, Inc., Chicago, IL).

## Results

As shown in Table 1, when comparing older ED users with major neurocognitive disorders to those without, older ED users with major neurocognitive disorders were older, less frequently living at home, and more often had home support, polypharmacy, and temporal disorientation (*P* ≤ 0.012). The reasons for ED visits significantly differed among older ED users with major neurocognitive disorders compared to those without. Those with major neurocognitive disorders less often visited EDs for organ failure and mobility disorders but more frequently for neuropsychiatric disorders and social issues (*P* < 0.001). The distribution of urgency levels was significantly different between groups (*P* < 0.05). Mean ER^2^ scores were higher in older ED users with major neurocognitive disorders (*P* < 0.001). There were more high-risk patients and fewer at the low risk level among participants with major neurocognitive disorders (P <0.001). The sensitivities of both ER^2^'s high risk level and temporal disorientation were high (≥0.91). Specificity, PPV, LR+, and AROC were higher for the temporal disorientation item compared to ER^2^'s high risk level, whereas the highest sensitivity, LR-, and NPV were obtained with the ER^2^ high risk level (Table 2). In addition, both AROC were high (0.71 for ER^2^'s high-risk level and 0.82 for ER^2^ temporal disorientation item). Logistic regressions showed that the odds ratios (OR) of ER^2^'s high risk level and of temporal disorientation for the diagnosis of major neurocognitive disorders were positive and significant (all *P*-values < 0.001), with the highest OR being reported for the temporal disorientation item in the unadjusted model [OR = 26.4 with 95% confidence interval (CI) = 17.7–39.3] (Table 3).

**Table 1:** Participant characteristics comparing patients with and without major neurocognitive disorder status (*n* = 999).

	**Major neurocognitive disorders**		*P*-value^*^
	**Present**	**Absent**	
	**(*n* = 356)**	**(*n* = 643)**	
Age (years), mean ± SD	86.6 ± 5.3	83.9 ± 5.5	**<0.001**
Female, *n* (%)	237 (66.4)	414 (64.4)	0.525
Living at home	133 (37.3)	432 (67.2)	**<0.001**
Home support^†^	314 (88.0)	398 (61.9)	**<0.001**
Polypharmacy^‡^	288 (80.7)	475 (73.9)	**0.012**
Use of walking aid^||^	572 (25.6)	433 (25.5)	0.935
Temporal disorientation^¶^	323 (90.5)	174 (27.1)	**<0.001**
**Reason for ED visit**, ***n*** **(%)**			
Organ failure^§^	157 (44.0)	449 (69.8)	**<0.001**
Mobility disorders^#^	62 (17.4)	122 (19.0)	**<0.001**
Neuropsychiatric disorders^**^	106 (29.7)	43 (6.7)	**<0.001**
Social issue	32 (9.0)	29 (4.5)	**<0.001**
**Canadian ED Triage and acuity scale**, ***n*** **(%)**			
Level 1–Resuscitation	15 (4.2)	6 (0.9)	**0.001**
Level 2–Emergent	81 (22.7)	200 (31.1)	**0.005**
Level 3–Urgent	187 (52.7)	338 (52.6)	0.955
Level 4–Less urgent	72 (20.2)	96 (14.9)	**0.034**
Level 5–Non-urgent	2 (0.7)	3 (0.5)	0.841
**ER** ^**2**^			
Score (/14), mean ± SD	10.3 ± 3.2	5.7 ± 4.1	**<0.001**
**Risk levels** ^**‡‡**^			
Low	14 (3.9)	280 (43.5)	**<0.001**
Moderate	2 (0.6)	19 (3.0)	**0.012**
High	340 (95.2)	344 (53.5)	**<0.001**
Hospital admission	172 (48.2)	298 (46.3)	0.578

*SD, Standard deviation; ED, Emergency department; ER^2^, Emergency Room Evaluation and Recommendation;*

* *, Comparisons based on unpaired t-tests or chi-square tests, as appropriate;*

† *, use of formal (health care or social services) and/or informal (family and/or friend) home support;*

‡ *, Number of medications taken daily ≥ 5;*

|| *, All categories of walking aids;*

¶ *, Inability to name current year and/or month;*

§ *, Defined as an acute organ decompensation;*

# *, Defined as gait and/or balance impairment and/or fall with or without fall-related injuries;*

** *, Defined as delirium, dementia, or behavioral disorders;*

†† *, Defined as the absence of symptoms of acute disease combined with an acute increase of the use of formal and/or informal home and social services leading to an inability to cope at home;*

‡‡ *, Low risk (score 0–3), Moderate risk (score 4–5), High risk (score ≥ 6); Significant P-values (i.e., <0.05) in bold*.

**Table 2:** Performance criteria of ER^2^'s high risk level and ER^2^'s temporal disorientation item for the diagnosis of major neurocognitive disorders (*n* = 999).

	**ER** ^**2**^	
	**High risk level**	**Item temporal disorientation**
Sensitivity	0.96	0.91
Specificity	0.47	0.73
Positive predictive value	0.59	0.65
Negative predictive value	0.95	0.93
Likelihood ratio of positive test	1.79	3.35
Likelihood ratio of negative test	10.34	7.87
Area under the receiver operating characteristic curve	0.71	0.82

*ER^2^, Emergency Room Evaluation and Recommendation*.

**Table 3:** Regressions showing the association of the diagnosis of major neurocognitive disorders (dependent variable) with ER^2^'s high risk level and ER^2^'s temporal disorientation item (independent variables) (*n* = 999).

	**Model 1**			**Model 2**		
	**OR**	**[95%CI]**	***P*-Value**	**OR**	**[95%CI]**	***P*-Value**
ER^2^ high risk level	18.5	[10.9;31.2]	**≤0.001**	18.4	[10.0;33.9]	**≤0.001**
A ER^2^ temporal disorientation item	26.4	[17.7;39.3]	**≤0.001**	16.6	[10.9;25.2]	**≤0.001**

*ER^2^, Emergency Room Evaluation and Recommendation; ED, Emergency department; OR, Odds ratio; CI, Confidence interval; Model 1, unadjusted; Model 2, adjusted on age, sex, living at home; use of home support, polypharmacy, use of walking aid, Canadian ED Triage and acuity scale, and reasons for emergency department visit; significant P-values (i.e., <0.05) in bold*.

## Discussion

The findings show that ER^2^ may be used to screen older ED users for their risk of major neurocognitive disorders. Both the ER^2^ high risk level and the “temporal disorientation” item included in ER^2^ were able to properly detect these disorders upon arrival to the ED. The “temporal disorientation” item alone showed superior performance to ER^2^'s high risk level to predict a major neurocognitive disorder diagnosis, while ER^2^'s high risk level was even more sensitive. Additionally, the findings confirm the high prevalence of major neurocognitive disorders of 36.5% in older ED users. In sum, we found that the inability to give the month and/or year upon arrival to the ED may be useful to screen older ED users for major neurocognitive disorders and easy to use in such busy area.

When it comes to taking care of older ED users, ED staff are concerned with being able to identify the right patients (i.e., those most at risk of adverse outcomes) at the right time (i.e., as soon as possible) and to introduce the right interventions (i.e., those most appropriate to patient health and functional conditions) with the objective of reducing the occurrence of undesirable events (6–8). The best way to prevent or minimize these undesirable events is screening older patients as soon as possible (i.e., earlier in the ED care plan) to establish those at the highest risk and who are candidates for timely and appropriate interventions (6, 10, 13). Major neurocognitive disorders are underdiagnosed by clinicians and underreported by patients and families, especially in EDs, whereas cognitive impairment/delirium are geriatric conditions that must be identified to designed a tailored care plan (1). Only about half of individuals who meet the criteria for major neurocognitive disorders are diagnosed by a clinician (1, 11). Furthermore, the diagnosis of major neurocognitive disorders is often delayed, with more older patients diagnosed at advanced stages of neurocognitive disorders, rather than at their onset (4, 9). As such, temporal disorientation is frequently observed in this population. This may explain why temporal disorientation can be an efficient way to screen older ED users for major neurocognitive disorders. Firstly, this emphasizes that patients with major neurocognitive disorders at more advanced stages are at greater risk of undesirable events compared to those who are at the onset of these disorders. Thus, it is important to screen the subgroup of patients visiting EDs with more advanced impairments, who are more at risk compared to those at the onset of major neurocognitive disorders (12). Second, an inability to give the month and/or year may be a marker of two types of cognitive impairment: major neurocognitive disorders at moderate to severe stages, as well as delirium (13). Delirium is highly frequent in patients with major neurocognitive disorders, especially in new environments and settings of acute disease, which are two conditions characterizing the ED (15, 16). Third, delirium is a red flag for the underlying presence of major neurocognitive disorders in patients who are otherwise undiagnosed (16). Thus, temporal disorientation should trigger a cognitive assessment, which should consist of two separate processes. First, the diagnosis of delirium must promptly be examined using standardized testing like the Confusion Assessment Method. Second, if the Confusion Assessment Method test is negative, the diagnosis of major neurocognitive disorders may be discussed later in the care process, following ED discharge. Regardless of the clinical diagnosis (i.e., delirium vs. major neurocognitive disorders), it is mandatory to immediately initiate interventions to limit the occurrence of undesirable events (7, 13). This last point highlights the advantage of choosing screening tests for cognitive impairment (i.e., delirium vs. major neurocognitive disorders) that are more sensitive than specific, especially as there are few potential adverse effects to interventions aimed to avoid the occurrence of undesirable events. Therefore, we suggest that it would be favorable to use the temporal disorientation item of ER^2^ as a screening tool for major neurocognitive disorders. Indeed, even if this item may be overly sensitive by classifying patients as having major neurocognitive disorders when they do not, there is a net benefit to introducing interventions that will improve the care of those who are cognitively impaired.

A good screening tool should be based on reliable (i.e., objective, standardized, and communicable) and valid clinical information, which can easily be obtained in busy EDs and used by all ED staff (17). Both ER^2^ and its “temporal disorientation” item meet these criteria. Recently, we demonstrated the usability of ER^2^ in EDs and its compatibility with daily ED practice (12). For instance, we reported that the mean time to collect and record ER^2^ items into a patient's digital chart was 3 min at bedside. It has been shown that, for a screening tool to be considered usable in the ED setting, personnel must be able to complete it in <5 min (18). Moreover, a recent systematic review showed that most tools do not consider usability in their development (19). It is therefore important to note that the ER^2^ study was classified as a clinical quality improvement program for older ED users. This choice was made to consider the *real-life* conditions of ED practice, ensuring that ER^2^ could feasibly be integrated into daily practice by ED nurses.

We observed a high prevalence of major neurocognitive disorders in older ED users, which is consistent with previous studies' findings of up to 30% (1–5).

There are limitations to consider in our study. Firstly, the study involved a single center, raising the possibility that the studied population may not be representative of all older ED users. This limits the external validity of the results. Secondly, the diagnosis of major neurocognitive disorders was established retrospectively based on information collected in participant's electronic medical records, rather than by an exhaustive examination. As a result, the processes leading to diagnoses of major neurocognitive disorders may be variable and more likely underestimated. Third, the present study examined major neurocognitive disorders as a binary endpoint and, thus, their severity and type could not be considered.

In conclusion, our results suggest that ER^2^ and, especially, its temporal disorientation item may be used to screen for major neurocognitive disorders in older ED users. There is a need to confirm these results using multicentre observational cohort studies in additional Canadian hospitals.

## Data Availability Statement

Data and materials are available on request. Request should be sent to the corresponding author: Olivier Beauchet, Research Centre of the Geriatric University institute of Montreal, Montreal, QC, Canada; E-mail: olivier.beauchet@umontreal.ca. All requests need a cover letter explaining the objective, justification and the referent ethic committee.

## Ethics Statement

The Jewish General Hospital (McGill University, Quebec, Canada) Research Ethics Committee approved the study. The Ethics Committee waived the requirement of written informed consent for participation. Verbal informed consent was obtained for all participants.

## Author Contributions

OB: principal investigator, study conception and design, obtaining funding, recruitment of participants, analyzing data, drafting first version of the manuscript, and approval of the final manuscript. MA and CL: study design, recruitment of participants, analyzing data, drafting first version, and approval of the final manuscript. LC-B, JL, and GA: revising the manuscript critically for important intellectual content and approval of the final manuscript. All authors contributed to the article and approved the submitted version.

## Funding

The study was financially supported by private donations (Oberfeld family) and the Foundation of the Jewish General Hospital (Montreal, QC, Canada). The funding sources have no involvement in study design; in the collection, analysis and interpretation of data; in the writing of the manuscript; and in the decision to submit the article for publication.

## Conflict of Interest

The authors declare that the research was conducted in the absence of any commercial or financial relationships that could be construed as a potential conflict of interest.

## Publisher's Note

All claims expressed in this article are solely those of the authors and do not necessarily represent those of their affiliated organizations, or those of the publisher, the editors and the reviewers. Any product that may be evaluated in this article, or claim that may be made by its manufacturer, is not guaranteed or endorsed by the publisher.

## Footnotes

^1^Available online at: https://data.oecd.org/healtheqt/hospital-beds.htm
